# Medication-Seeking Behaviors and Correlated Characteristics of Zolpidem Users in Taiwan—A Shared Patient Network Analysis

**DOI:** 10.3390/healthcare12060660

**Published:** 2024-03-14

**Authors:** Yi-Ju Pan, Sheng-Hsuan Chang, Wei-Chen Lee, Yu-Chun Chen

**Affiliations:** 1 Department of Psychiatry, Far Eastern Memorial Hospital, New Taipei City 220, Taiwan; yjpan@mail.femh.org.tw (Y.-J.P.); 101311144@gms.tcu.edu.tw (W.-C.L.); 2 Department of Chemical Engineering and Materials Science, Yuan Ze University, Taoyuan City 320, Taiwan; 3 Institute of Hospital and Health Care Administration, National Yang Ming Chiao Tung University, Taipei 112, Taiwan; caroline80731@gmail.com; 4 School of Medicine, College of Medicine, National Yang Ming Chiao Tung University, Taipei 112, Taiwan; 5 Department of Family Medicine, Taipei Veterans General Hospital, Taipei 112, Taiwan; 6 Big Data Center, Taipei Veterans General Hospital, Taipei 112, Taiwan

**Keywords:** sedative–hypnotic drug, zolpidem, medication-seeking behavior

## Abstract

Increasing insomnia signals a public health problem, alongside rising zolpidem use. This study investigates the factors behind the disproportionate rise in zolpidem prescriptions in Taiwan. It aims to identify the determinants of high-dose zolpidem users in Taiwan’s Yilan County and employ an innovative approach to outline their medication-seeking patterns, using Taiwan’s healthcare database. The associations between sociodemographic and clinical factors and low-dose and high-dose users were analyzed using multiple logistic regression. Social network analysis was employed to explore medication-seeking behavior among these user groups across different healthcare institutions. Of our 5290 participants, 22.82% are high-dose users. This study found that males face a 1.33-fold higher risk and that having chronic diseases is a major risk factor, contributing to a more than four-times higher risk (adjusted OR = 4.27, 95% CI 1.55–11.70) of being a high-dose user of zolpidem. A social network analysis showed a higher density (0.52) for high-dose users, revealing their frequent visits, for zolpidem, to different healthcare institutions. Psychiatrists have a central role in both low-dose and high-dose user networks, with a greater influence on low-dose users (64.4) than high-dose users (32.2). In sum, patients seeking high doses of zolpidem are driven by personal factors. Future efforts should include regulated dispensing, public health education, and specialized training for healthcare professionals on drug addiction.

## 1. Introduction

Insomnia is a significant public health issue affecting a considerable portion of the population at various points in their lives, leading to a decreased quality of life, increased absenteeism, disability, and higher healthcare costs [1]. Previous surveys have indicated that one third to one half of the general population may experience symptoms of insomnia at least once in their lifetime, with about 70% of these cases following a chronic course over a 1-year follow-up [2,3]. The prevalence of insomnia in Taiwan has been documented in a series of national surveys, showing its increase over time, which has correspondingly led to a disproportionate increase in the prescription of medication for its treatment, highlighting concerns regarding overmedication, abuse/dependence, and the potential adverse effects of the long-term use of sedative hypnotics [4,5].

Zolpidem, as the most frequently prescribed non-benzodiazepine sedative–hypnotic agent in many countries, has seen a notable increase in its prescription in Taiwan [6,7]. Despite the preference for it due to its lower risk of causing daytime sleepiness, zolpidem is associated with severe adverse effects like sleepwalking, hallucinations, heightened suicidality, and complex behaviors [8]. Additionally, it poses risks of abuse and addiction, as indicated by an increasing trend in “Suspect Prescriptions Possibly Indicating Abuse” [9]. Specifically, in the elderly population in Taiwan, there has been a significant increase in the use of z-hypnotics, with zolpidem accounting for a substantial portion of these prescriptions [10]. 

A significant shift in z-hypnotic prescriptions from medical centers to local clinics has been observed in Taiwan, with most prescriptions in 2010 coming from non-psychiatrist physicians [10]. Additionally, sedative hypnotics were prescribed in 51.7% of psychiatric outpatient visits in 2012, with zolpidem being the most commonly prescribed [11]. This shift indicates that there is a need to explore the factors behind the increasing prescription of zolpidem, given its potential for overmedication. Crucially, zolpidem is a controlled medication in Taiwan, not available over the counter, and is under stringent regulation in terms of its cumulative usage in a centralized system. This regulation is a critical step towards mitigating the risks associated with its potential for abuse and addiction, reflecting the government’s efforts to monitor its distribution and usage closely.

A social network analysis may be needed to investigate in this complex inter-relationship between patient characteristics, medication-seeking behaviors, and prescriptions from physicians of different specialties, as well as from different levels of healthcare institutions. In other health research domains, social network analysis has shown that obesity [12], smoking behaviors [13], and depressive symptoms [14] may spread in social networks in a quantifiable and discernable pattern that depends on the nature of the social ties. However, owing to the rarity of social network data in the context of physician behaviors and the patient’s use of healthcare services, another network concept—shared patients—was adopted in this domain of interest, connecting physicians, healthcare providers, or institutions through their shared-patient networks [15]. By employing a shared-patient network, existing evidence was able to show that physicians tend to share patients with other physicians with similar physician-level and patient-panel characteristics [16] and variations in the patient-sharing networks of physicians were found to differ in terms of healthcare patterns and the costs of their affiliated healthcare institutions or hospitals [17]. Conceptually, “shared patients” are formed from a bipartite network of healthcare providers and patients. Structurally, the healthcare provider is structured as a node and the shared patients are the ties. The density of the ties (shared patients) may reflect the intensity of the link between healthcare providers (or institutions) and their betweenness centrality may reflect the influence of individual healthcare providers (or institutions) on the investigated network. 

Therefore, the current study aimed to employ a shared-patient network analysis of physicians/institutions within a cohort of Taiwanese people who lived in Yilan County, were 40–80 years old, and had physician encounters with zolpidem prescriptions in 2008 to examine the differences in the patient characteristics which contribute to a higher use of zolpidem, as well as to explore the different network patterns of medication-seeking behaviors between high-dose users and low-dose users of zolpidem, as well as across different levels of healthcare. Furthermore, by employing this innovative research method, the aim was to depict the patient-sharing network of physicians/institutions in the Yilan County of Taiwan using measurements of density, in-degree, and betweenness centrality. This approach was intended to explore the influences of distinct types of physicians/institutions on the network of medication-seeking behaviors of zolpidem users over a one-year observational period.

## 2. Materials and Methods

### 2.1. Setting

This study was conducted in accordance with the Declaration of Helsinki and approved by the Research Ethics Review Committee of the Far Eastern Memorial Hospital of Taiwan (103179-F). Taiwan has a population of approximately 23,000,000. Healthcare institutions in Taiwan are classified by the Ministry of Health and Welfare into different levels, including medical centers, regional hospitals, district hospitals, psychiatric hospitals, and clinics. Each level of hospital is assigned distinct care responsibilities and roles. National Health Insurance (NHI) in Taiwan is a single-payer compulsory social insurance plan that centralizes the disbursement of healthcare funds and guarantees equal access to healthcare for all citizens. In 2008, a total of 22.92 million individuals were covered under Taiwan’s NHI program, with a coverage rate around 99% [18]. The National Health Insurance Research Database (NHIRD) comprises the NHI data on the healthcare utilization of insured residents, including their expenditures, medical procedures, and treatments, as well as the basic characteristics of patients, providers, and physicians. Until 2016, the International Classification of Diseases, Ninth Revision, Clinical Modification (ICD-9-CM) was used in the NHIRD [19].

### 2.2. Study Population

In this study, we conducted a comprehensive analysis using secondary data, thanks to special authorization obtained from the National Health Insurance (NHI) to access the National Health Insurance Research Database (NHIRD). This authorization allowed us to examine data pertaining to all individuals in Yilan County who were prescribed sleeping pills at any time between 2006 and 2009. It is crucial to highlight that this research is not based on a sampled population. Instead, it covers the entire population of Yilan County who met the criteria during the specified period.

The selection process began with determining individuals’ place of residence across various urbanization levels and age groups, achieved by integrating their insurance classification, hospital visit locations, and insurance registration data. The method employed to infer the proximity of outpatient services to the insured place involved using longitude and latitude to ensure a straight-line distance of 50 km or less, thereby aligning outpatient service usage with the likely place of residence of the insured in Yilan County [20].

This study targeted individuals aged 40 to 80 living in Yilan County. Those who had a record of insurance withdrawal in 2008 and no subsequent insurance registration in 2009 were presumed deceased and excluded. A crucial criterion for inclusion was a prescription history of zolpidem: individuals who received prescriptions at least four times in 2007 and at least once in 2008 were selected. These criteria aimed to focus on chronic, long-term users of zolpidem, rather than short-term users who might not have stable prescribing patterns and could still be moving between health providers. These individuals were thus classified as non-initial users for the year 2008, forming the final cohort for the study.

The dosage standard for zolpidem was set at 10 mg per day, equivalent to 1 Defined Daily Dose (DDD) as per the World Health Organization (WHO). The DDD is the assumed average maintenance dose per day for a drug used for its main indication in adults. This study referenced WHO guidelines to categorize individuals into low-dose users (an annual consumption ≤ 360 tablets) and high-dose users (an annual consumption > 360 tablets), thereby enabling a focused analysis on the prescribing network of zolpidem and its usage patterns among chronic users within the selected demographic.

### 2.3. Covariates

Data on age, gender, insurance premium level, possession of a catastrophic illness certification (which Taiwan’s National Health Insurance [NHI] provides to patients with severe, long-term illnesses, exempting them from medical co-payments for conditions such as cancer, chronic diseases, and rare illnesses), and the presence of chronic diseases, as defined by Taiwan’s NHI, were extracted for the study population [21]. Additionally, the records of outpatient visits between 2006 and 2009 in Yilan County, where sleeping pills were prescribed according to Clinical Classifications Software (CCS) criteria, were collected. This study also screened for the top 19 reasons for and other causes of visits in the outpatient records of the study population. Furthermore, data on the prescription of zolpidem across various healthcare institutions, including regional hospitals, district hospitals, and clinics, were gathered. The analysis then included calculating the proportion of prescriptions issued by psychiatrists compared to other physicians.

### 2.4. Data Analysis

A cross-sectional study design was used to track the study population of low-dose zolpidem users and high-dose zolpidem users from 1 January to 31 December 2008. The groups were analyzed via multiple logistic regression; through stepwise regression, the covariates of the study group included age, gender, insurance premium level, possession of a catastrophic illness certification, the presence of chronic diseases, and the prescription ratio of zolpidem at different levels of healthcare. Furthermore, the top 19 reasons for and other causes of visits in the study population’s outpatient records were explored, and their correlation with both low-dose users and high-dose users of zolpidem was examined. The strength of the correlation was related to the adjusted odds ratio (aOR) and 95% confidence interval (CI).

A network visualization method was used to explore the network of medication-seeking behaviors of low-dose users and high-dose users of zolpidem across different levels of healthcare institutions. It was assumed that any two healthcare institutions were related based on the patients’ medication-seeking behaviors. Therefore, in this network diagram, the healthcare institutions are presented as nodes (Vertices), with their level distinguished by color: regional hospitals (red), district hospitals (green), clinics (blue), psychiatric hospitals (brown), and psychiatrists (purple). The frequency count of patients’ medication-seeking behaviors was logarithmically transformed, resulting in varying line thicknesses and opacities for the “Edges” connecting healthcare institutions. Three indicators were used to explore the important information about patients’ medication-seeking behavior, as follows:

Density: Density is used to analyze the density of the network between medical institutions due to a patient’s medication-seeking behavior. If this is closer to 1, it means that patients have a high density of medication-seeking behaviors between various medical institutions.

In-Degree: Each medical institution will generate an internal connection, indicating the number of sets of patients who have been to other medical institutions in the network before going to that hospital. For example, if Hospital A’s number of internal connections is 20, that means that the patient has visited 20 other medical institutions in the network before arriving at Hospital A.

Betweenness Centrality: This mainly measures the importance of a medical institution in the network between any two medical institution paths. If a medical institution exhibits a higher betweenness centrality in the network, it signifies that it plays a more critical role as a bridge or critical point within that network [21]. 

The SQL server 2024 was used for debugging and data linking. All statistical analyses were performed using SPSS version 22.0 (IBM, Armonk, NY, USA). Finally, NodeXL [22] was used for network analysis.

## 3. Results

The land area of Yilan County is 2143.6251 square kilometers, accounting for 6.02% of the total area of Taiwan. In 2008, its population was 460,902. Among all its administrative regions, Yilan City had the largest population, 95,874, accounting for 20.80% of the total population of Yilan County, followed by Luodong. There were 73,722 people (16.00%) in Datong Township, 5815 people in Datong Township, and 5842 people in Nan’ao Township, the latter two accounting for only 1.26% and 1.27%, respectively [23]. 

**Figure 1:**
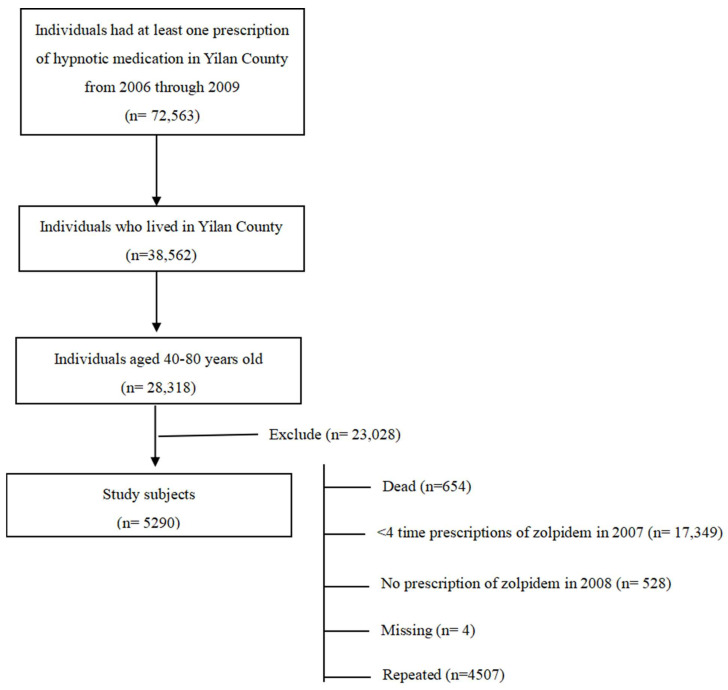
Flowchart for study subject selection and recruitment.

From 2006 through 2009, a total of 72,563 individuals in Yilan County had at least one prescription for a sedative–hypnotic medication. Among them, 38,562 individuals who lived in Yilan County were identified using a method that combined their insurance classification, the locations of their hospital visits, and their insurance registration. Then, 28,318 of them were found to be aged 40–80 years old. After further excluding those who were deceased (n = 654), those who had fewer than four prescriptions of zolpidem in 2007 (n = 17,349), those who had no prescription of zolpidem in 2008 (n = 9528), missing cases (n = 4), and repeated cases (n = 4507), the final study cohort included 5290 study subjects who received at least one prescription of zolpidem in 2008 (Figure 1).

The study population’s characteristics are revealed in Table 1. It shows that 63.95% (n = 3383) of the study subjects were females. Their mean age was 60 years old (mean age of females = 59.6; mean age of males = 60.7). According to the age distribution of the patients, 1518 individuals (28.70%) were aged 51–60 years old, the largest group, followed by 1310 individuals (24.76%) aged 40–50 years old, and 1237 individuals (23.38%) aged 61–70 years old, respectively. Of the study subjects, 22.80% had a catastrophic illness certification. There were 2196 individuals (58.49%) obtaining zolpidem from clinics, 2311 individuals (43.69%) obtaining it from regional hospitals, and 533 individuals (10.08%) obtaining it from district hospitals, respectively.

Our analysis of low-dose users vs. high-dose users is revealed in Table 2. The proportion of low-dose users was 77.18% (n = 4083) and the proportion of high-dose users was 22.82% (n = 1207), respectively. The proportion of female low-dose users is 1.7 times higher than of males (64.0% vs. 36.0%), and, similarly, the proportion of female high-dose users is also 1.7 times higher than that of males (63.7% vs. 36.3%). Regarding the distribution of age groups, the largest percentage of low-dose users was the group of individuals who were aged 51–60 years old, at 1160 individuals (28.4%). For high-dose users, their largest percentage was the group individuals who were aged 40–50 years old, at 385 individuals (31.9%), followed by the age group of 51–60, at 358 individuals (29.7%). According to the logistic regression analysis, the correlation between being male and being a high-dose user is significant (aOR = 1.33, 95% CI = 1.10–1.60, *p* < 0.001), suggesting a 1.33-fold higher likelihood of men being high-dose users compared to their female counterparts. Regarding age, compared to individuals aged 71–80 years, those aged 40–50 years had a 1.63-fold higher risk of being high-dose users (aOR = 1.63, 95% CI = 1.32–2.01, *p* < 0.001) and those aged 51–60 years had a 1.23-fold higher risk of being high-dose users (aOR = 1.23, 95% CI = 1.00–1.51, *p* < 0.05), respectively. Additionally, individuals with a catastrophic illness certification or chronic diseases are significantly associated with being high-dose users. A low income is also significantly associated with high-dose users. Individuals with a monthly insurance premium less than or equal to NTD 20,000 had a 1.40-fold higher risk of being high-dose users compared to those with a premium higher than NTD 40,000. Individuals whose chief reasons for their visits include mood disorders, hypertension, other emotional disturbances, gastrointestinal diseases, coronary heart disease, or degenerative arthritis, all exhibited a higher risk of being high-dose users.

**Figure 2:**
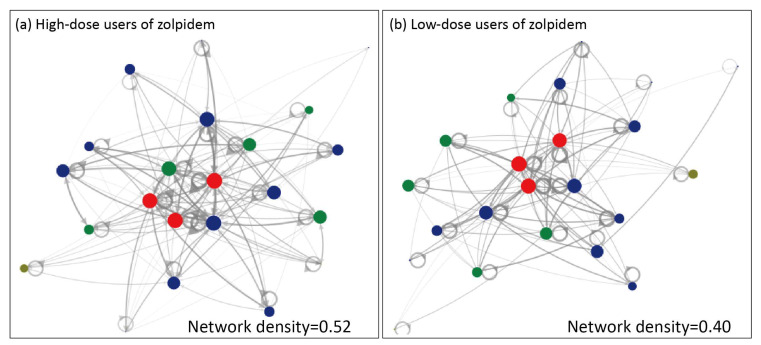
Network diagram of high-dose (**a**) and low-dose (**b**) users of zolpidem. The healthcare institutions are presented as nodes (Vertices), with their level distinguished by color: regional hospitals (red), district hospitals (green) and clinics (blue). Gray nodes denote healthcare institutes outside of Yilan county. The frequency count of patients’ medication-seeking behaviors was logarithmically transformed, resulting in varying line thickness and opacity for the Edges connecting healthcare institutions.

**Figure 3:**
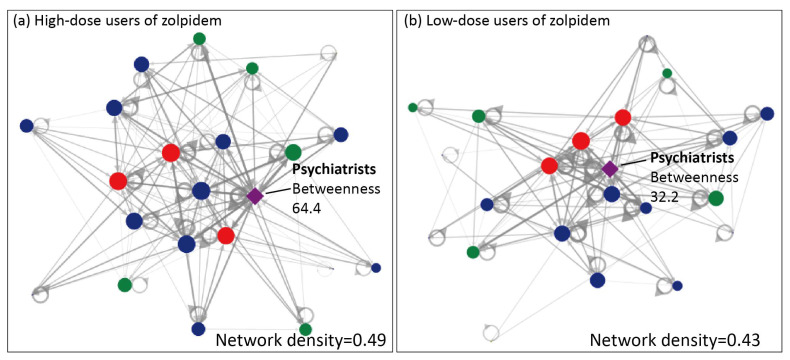
Network diagram of high-dose (**a**) and low-dose (**b**) users of zolpidem incorporating psychiatrists. The healthcare institutions are presented as nodes (Vertices), with their level distinguished by color: regional hospitals (red), district hospitals (green), clinics (blue), and psychiatrists (purple). Gray nodes denote healthcare institutes outside of Yilan county. The frequency count of patients’ medication-seeking behaviors was logarithmically transformed, resulting in varying line thickness and opacity for the Edges connecting healthcare institutions.

The results of the social network analysis are revealed in Figure 2 and Figure 3. It was found in Figure 2 that high-dose users had a higher density (density = 0.52) compared to low-dose users (density = 0.4). Regarding the betweenness centrality analysis, the ranking for the top three central positions of medical institutions differed between low-dose users and high-dose users. Moreover, clinic F did not rank within the top ten for low-dose users but secured the eighth position in in-degree among high-dose users. Its betweenness centrality also ranked seventh (Supplementary Tables S1 and S2).

The results of incorporating the role of psychiatrists into the social network analysis are revealed in Figure 3. Their density for high-dose users is 0.49, while for low-dose users it is 0.43. The ranking based on the in-degree analysis shows that the top position for low-dose users is occupied by psychiatrists (in-degree = 20), followed by other medical institutions. However, for high-dose users, the in-degree analysis showed their lower ranking for psychiatrists compared to that of low-dose users. When incorporating psychiatrists into the betweenness centrality analysis, the top three central positions for low-dose users are occupied by regional hospital B (betweenness centrality = 68.2), psychiatrists (betweenness centrality = 64.4), and regional hospital A (betweenness centrality = 30.7). In contrast, for high-dose users, the top three central positions are held by regional hospital A (betweenness centrality = 35.7), clinic A (betweenness centrality = 33.0), and psychiatrists (betweenness centrality = 32.2).

## 4. Discussion

The existing literature has ever-explored the impacts of social networks on substance abuse in adolescents [24], but very few studies have focused on the interrelationships between adult individuals’ use of prescribed sedative hypnotics and their related networks, and none of them have focused on a single prescribed medication such as zolpidem. 

This study provided new evidence on factors influencing the prescribing network of zolpidem in a cohort of residents of Yilan County who were aged 40–80 years old and had insomnia complaints. The present study revealed that having chronic diseases is a major risk factor, contributing to a more than four-times higher risk (adjusted OR = 4.27, 95% CI 1.55–11.70) of being a high-dose user of zolpidem. A prior Taiwanese study showed that patients with chronic diseases, particularly those with multiple health conditions, were more likely to display doctor shopping behaviors for zolpidem. [5] Taiwan’s NHI allows physicians to write refillable prescriptions for patients with chronic illnesses [25]. The time allowance of the refillable prescriptions is usually for 90 days at most. If zolpidem was prescribed with a refillable prescription, patients could easily obtain a large quantity of medication. It is therefore understandable that patients who have more diseases have a higher likelihood of going to different hospitals and repeatedly seeking help from doctors with different specialties. Additionally, the prescription drug is paid for by the NHI, and the patient does not need to pay out of pocket, which may also contribute to the problem of repeated prescriptions of zolpidem [5].

Among the top 19 reasons for and other causes of visits to healthcare centers for zolpidem prescriptions, we found that a person having a cardiovascular disease, gastrointestinal disease, or degenerative arthritis represented significantly increased risk of them being a high-dose user of zolpidem. A previous Taiwanese study on the prescriptions of z-hypnotics among elderly patients showed that around 15% of z-hypnotics were prescribed by psychiatrists, while most z-hypnotics were prescribed by physicians not specializing in psychiatry, which included physicians of internal medicine (28.3%), family medicine (21.7%), cardiology (12%), and neurology (9.7%) [10]. The distributions of physicians’ specialties across the prescriptions of zolpidem might partly reflect the distributions of the physical complaints of elderly patients who sought medical attention while simultaneously having insomnia complaints in Taiwan. The extent to which each physical illness may influence the onset or maintenance of insomnia symptoms also warrants further investigation.

The social network analysis showed that high-dose users of zolpidem had a higher density (density = 0.52) than low-dose users (density = 0.4), which suggests a more frequent medication-seeking behavior between different hospitals/clinics in high-dose users of zolpidem. Additionally, the in-degree analyses also revealed, when comparing the in-degree values of high-dose users and low-dose users within the same healthcare institutions, that the in-degree values of high-dose users are larger than those of low-dose users. This suggests a higher number of hospital/clinics visits before the indexed visit. Last but perhaps most importantly, the betweenness centrality measurements showed that several hospitals/clinics in this area of interest had stronger influences in the high-dose users’ network than in low-dose users’ network. The variations in the network measurements found between the low-dose users and high-dose users’ networks confirmed the existence of distinct roles and influences of different healthcare providers or institutions on the network of zolpidem prescriptions in Yilan County.

Our findings from the network measurements also confirmed the important role of psychiatrists in the network of zolpidem prescriptions. This is understandable given that having mood disorders was shown to be the single disease entity which had the highest risk of a sufferer being a high-dose user of zolpidem (adjusted OR = 3.93, 95% CI 3.27–4.73) in this study. In fact, insomnia may be the most common symptom in individuals with mental disorders. For instance, at least 80% of patients with a major depressive disorder have been shown to present insomnia-related symptoms [26]. Prior studies have also revealed that insomnia is strongly linked (odds ratios = 4.0–6.0) to poor mental and physical health, psychological distress, anxiety, depression symptoms, and somatic symptoms [27,28,29,30]. The social network analyses revealed that, based on our betweenness centrality analyses involving psychiatrists and healthcare institutions of any level, higher betweenness centrality values were observed in low-dose users (betweenness centrality = 64.4) compared to high-dose users (betweenness centrality = 32.2). This suggested that psychiatrists may have a stronger influence on the network of low-dose users of zolpidem than on the network of high-dose users. Therefore, psychiatrists may have a central role in the medication-seeking behavior of low-dose zolpidem users; however, for high-dose zolpidem users, the influencing role of psychiatrists is weakened. For patients using high doses of zolpidem, their medication-seeking behaviors seemed to be mainly driven by patient-related factors. In the future, efforts should be directed towards government-regulated dispensing systems for sedative–hypnotic drugs to prevent repetitive refills of sedative hypnotics; additionally, widespread public health education and specialized training for healthcare professionals across various specialties on prescribing sedative–hypnotic drugs, especially in terms of their knowledge related to drug addiction, are urgently needed. There is also a need to further strengthen the role of psychiatrists within the healthcare network to address and improve the issue of sedative–hypnotic drug abuse.

This study has several limitations. First, it is a cross-sectional study. Our analysis was based solely on outpatient data from the year 2008, without considering hospitalized patients. Future research may concurrently investigate the usage of zolpidem among hospitalized patients and outpatients, employing a longitudinal study design. This could offer a more thorough understanding of the overall trends in zolpidem misuse in Taiwan. Second, the study population was restricted to individuals aged 40–80 residing in Yilan County. However, some insured individuals may seek medical care outside Yilan, potentially leading to discrepancies in the calculation of insurance and residency locations. Third, the NHI database lacks individual information such as accurate socioeconomic status and education levels. Insurance premium levels were used as an indicator of socioeconomic status, which may deviate from individuals’ actual real-life situations. Lastly, although the NHI database encompasses over 96% of the population of Taiwan seeking medical care, individuals seeking zolpidem may obtain the drug for purposes other than personal use or may purchase it through self-payment, which may not be reflected in this database.

## 5. Conclusions

In conclusion, males; those aged 40–60 years old; individuals with chronic diseases, possession of a catastrophic illness certification, or who are economically disadvantaged; and individuals whose chief reasons for healthcare visits include mood disorders, hypertension, other emotional disturbances, gastrointestinal diseases, coronary heart disease, and degenerative arthritis, all exhibit a higher risk of being high-dose users of zolpidem. High-dose users of zolpidem exhibited a higher “density” of medication-seeking behaviors than low-dose users, which may refer to more frequent doctor shopping behaviors and medication refills across different healthcare institutions. Psychiatrists were shown to have an important role in the zolpidem-seeking network in Yilan County. However, the central role of psychiatrists was weakened in the network of high-dose users compared to that of low-dose users. Through a social network analysis, the presence of zolpidem-seeking behaviors was visually observed. These distinct processes were varied between high-dose and low-dose users, which highlighted the presence of differences in their medical needs. Our findings provide valuable insights for government entities and policymakers, with the hope that more effective strategies will be developed in the future to address zolpidem-related medication-seeking behaviors.

## Figures and Tables

**Table 1:** Characteristics of zolpidem users in 2008.

	Zolpidem Users (n = 5290)	%
Gender		
Female	3383	63.95%
Male	1907	36.05%
Age		
40–50	1310	24.76%
51–60	1518	28.70%
61–70	1237	23.38%
71–80	1225	23.16%
Monthly insurance premium		
≤20,000	999	18.88%
20,001–40,000	3870	73.16%
>40,000	421	7.96%
Catastrophic illness certification		
Yes	1206	22.80%
No	4084	77.20%
Chronic disease		
Yes	5171	97.75%
No	119	2.25%
Level of healthcare institution		
Regional hospital	2311	43.69%
District hospital	533	10.08%
Clinics	2196	58.49%

**Table 2:** Comparisons of demographic characteristics (top half) and reasons for healthcare institution visits (bottom half) between low-dose users and high-dose users of zolpidem.

	Low-Dose Users		High-Dose Users		Adjusted Odds Ratio (aOR)			
	n	N%	n	N%	aOR	95% CI	*p*	Sig
Gender								
Female	2614	64.0%	769	63.7%	1.00			
Male	1469	36.0%	438	36.3%	1.33	1.10–1.60	0.003	**
Age								
40–50	925	22.7%	385	31.9%	1.63	1.32–2.01	<0.001	***
51–60	1160	28.4%	358	29.7%	1.23	1.00–1.51	0.046	*
61–70	999	24.5%	238	19.7%	1.04	0.84–1.29	0.719	
71–80	999	24.5%	226	18.7%	1.00			
Monthly insurance premium (NTD)								
≤20,000	722	17.7%	277	22.9%	1.40	1.18–1.66	<0.001	***
20,001–40,000	334	8.2%	87	7.2%	0.84	0.64–1.10	0.202	
>40,000	3027	74.1%	843	69.9%	1.00			
Catastrophic illness certification								
Yes	744	18.2%	462	38.3%	2.08	1.78–2.43	<0.001	***
No	3339	81.8%	745	61.7%	1.00			
Chronic disease								
Yes	3968	97.2%	1203	99.7%	4.27	1.55–11.70	0.005	**
No	115	2.8%	4	0.3%	1.00			
Reasons for visits ^a^								
Mood disorder								
Yes	978	24.0%	653	54.1%	3.93	3.27–4.73	<0.001	***
No	3105	76.0%	554	45.9%	1.00			
Hypertension								
Yes	858	21.0%	228	18.9%	1.31	1.09–1.56	0.004	**
No	3225	79.0%	979	81.1%	1.00			
Other emotional disturbances								
Yes	488	12.0%	192	15.9%	1.77	1.45–2.15	<0.001	***
No	3595	88.0%	1015	84.1%	1.00			
Gastrointestinal diseases								
Yes	294	7.2%	141	11.7%	1.64	1.30–2.06	<0.001	***
No	3789	92.8%	1066	88.3%	1.00			
Coronary heart disease								
Yes	386	9.5%	128	10.6%	1.80	1.43–2.27	<0.001	***
No	3697	90.5%	1079	89.4%	1.00			
Degenerative arthritis								
Yes	297	7.3%	102	8.5%	1.56	1.21–2.02	0.001	**
No	3786	92.7%	1105	91.5%	1.00			

^a^ Reasons for visits: anxiety (n = 72), mood disorder (n = 69), hypertension (n = 98), common cold (n = 126), other emotional disturbances (n = 74), diabetes mellitus (n = 49), dizziness (n = 93), hyperlipidemia (n = 53), musculoskeletal disorders (n = 211), back pain (n = 205), headache (n = 84), gastrointestinal disease (n = 155), coronary heart disease (n = 101), degenerative arthritis (n = 203), chronic obstructive pulmonary disease (n = 127), arrhythmia (n = 106), bronchitis (n = 125), other gastric diseases (n = 141), gastric ulcer (n = 139), and others (n = 259). Abbreviations: CI, confidence interval; Sig, significance. * *p* < 0.05, ** *p* < 0.01, *** *p* < 0.001.

## Data Availability

There are ethical or legal restrictions on sharing the de-identified dataset, as established by the research ethics review committee.

## Supplementary Materials

The following supporting information can be downloaded at https://www.mdpi.com/article/10.3390/healthcare12060660/s1, Table S1: social network analysis of healthcare institutions in low-dose users and high-dose users of zolpidem; Table S2: social network analysis of both healthcare institutions and psychiatrists in low-dose users and high-dose users of zolpidem.
