# Supplementary material for: Medication-Seeking Behaviors and Correlated Characteristics of Zolpidem Users in Taiwan—A Shared Patient Network Analysis
Source: Healthcare (Basel). 2024 Mar 14;12(6):660. doi: 10.3390/healthcare12060660 (PMC10970090; doi:10.3390/healthcare12060660)
Supplement: Supplementary file 1 [file healthcare-12-00660-s001.zip › healthcare-2884175-supplementary.pdf]

**Supplementary Table S1.** Social network analysis for healthcare institutions in low-dose users and high-dose users of zolpidem

Low-dose users

| Density：0.40 | | | | |
|---|---|---|---|---|
| Ranking | | IN-Degree | | Betweenness Centrality |
| 1 | Regional hospital A | 19 | Regional hospital B | 89.2 |
| 2 | Regional hospital B | 17 | Regional hospital A | 60.1 |
| 3 | Regional hospital C | 17 | Clinic A | 30.5 |
| 4 | Clinic A | 16 | Regional hospital C | 29.3 |
| 5 | Clinic B | 15 | Clinic B | 18.5 |
| 6 | Clinic C | 11 | Clinic E | 8.3 |
| 7 | District hospital A | 10 | District hospital A | 6.7 |
| 8 | District hospital B | 9 | Clinic C | 4.6 |
| 9 | District hospital C | 9 | District hospital B | 4.3 |
| 10 | Clinic D | 9 | District hospital C | 4.3 |

High-dose users

| Density：0.52 | | | | |
|---|---|---|---|---|
| Ranking | | IN-Degree | | Betweenness Centrality |
| 1 | Regional hospital A | 22 | Regional hospital A | 42.7 |
| 2 | Clinic A | 21 | Regional hospital B | 30.6 |
| 3 | Regional hospital C | 20 | Clinic A | 30.5 |
| 4 | Regional hospital B | 20 | Regional hospital C | 25.5 |
| 5 | Clinic B | 18 | Clinic B | 20.8 |
| 6 | District hospital A | 17 | District hospital A | 20.1 |
| 7 | District hospital D | 13 | Clinic F | 8.5 |
| 8 | Clinic F | 12 | District hospital B | 6.4 |
| 9 | Clinic G | 12 | Clinic G | 5.3 |
| 10 | Clinic C | 12 | District hospital D | 4.9 |

**Supplementary Table S2.** Social network analysis for both healthcare institutions and psychiatrists in low-dose users and high-dose users of zolpidem

Low-dose users

| Density：0.43 | | | | |
|---|---|---|---|---|
| Ranking | | IN-Degree | | Betweenness Centrality |
| 1 | Psychiatrists | 20 | Regional hospital B | 68.2 |
| 2 | Regional hospital A | 18 | Psychiatrists | 64.4 |
| 3 | Regional hospital B | 16 | Regional hospital A | 30.7 |
| 4 | Clinic A | 15 | Regional hospital C | 23.0 |
| 5 | Clinic B | 15 | Clinic A | 18.8 |
| 6 | Regional hospital C | 15 | Clinic B | 16.7 |
| 7 | Clinic C | 12 | District hospital A | 10.7 |
| 8 | Clinic G | 10 | Clinic E | 9.00 |
| 9 | Clinic F | 10 | Clinic C | 5.7 |
| 10 | District hospital A | 10 | Clinic D | 3.1 |

High-dose users

| Density：0.49 | | | | |
|---|---|---|---|---|
| Ranking | | IN-Degree | | Betweenness Centrality |
| 1 | Clinic A | 20 | Regional hospital A | 35.7 |
| 2 | Psychiatrists | 20 | Clinic A | 33.0 |
| 3 | Regional hospital A | 19 | Psychiatrists | 32.2 |
| 4 | Regional hospital B | 18 | Regional hospital B | 27.4 |
| 5 | Clinic B | 17 | Clinic B | 22.6 |
| 6 | Regional hospital C | 14 | Regional hospital C | 16.9 |
| 7 | District hospital A | 14 | Clinic F | 11.9 |
| 8 | Clinic F | 13 | District hospital A | 10.7 |
| 9 | Clinic G | 12 | Clinic G | 7.4 |
| 10 | Clinic C | 12 | Clinic C | 4.3 |
